# Multifactorial Scores and Biomarkers of Prognosis of Acute Pancreatitis: Applications to Research and Practice

**DOI:** 10.3390/ijms21010338

**Published:** 2020-01-04

**Authors:** Pedro Silva-Vaz, Ana Margarida Abrantes, Miguel Castelo-Branco, António Gouveia, Maria Filomena Botelho, José Guilherme Tralhão

**Affiliations:** 1 Health Sciences Research Centre, University of Beira Interior (CICS-UBI), 6200-506 Covilhã, Portugal; mcbranco@fcsaude.ubi.pt; 2 General Surgery Department, Hospital Local de Saúde de Castelo Branco, 6000-085 Castelo Branco, Portugal; agouveia@ulscb.min-saude.pt; 3 Faculty of Health Sciences, University of Beira Interior, 6200-506 Covilhã, Portugal; 4 Coimbra Institute for Clinical and Biomedical Research (iCBR) area of Environment Genetics and Oncobiology (CIMAGO), Faculty of Medicine, University of Coimbra, 3000-548 Coimbra, Portugal; mabrantes@fmed.uc.pt (A.M.A.); mfbotelho@fmed.uc.pt (M.F.B.); jglrt@hotmail.com (J.G.T.); 5 Faculty of Medicine, University of Coimbra, 3000-548 Coimbra, Portugal; 6 Biophysics and Biomathematics Institute, IBILI-Faculty of Medicine of University of Coimbra, 3000-348 Coimbra, Portugal; 7 Surgery Department, Centro Hospitalar e Universitário de Coimbra (CHUC), University Hospital, Faculty of Medicine, 3000-075 Coimbra, Portugal

**Keywords:** acute pancreatitis, severity, prognostic, multifactorial scoring system, biomarker

## Abstract

Acute pancreatitis (AP) is a severe inflammation of the pancreas presented with sudden onset and severe abdominal pain with a high morbidity and mortality rate, if accompanied by severe local and systemic complications. Numerous studies have been published about the pathogenesis of AP; however, the precise mechanism behind this pathology remains unclear. Extensive research conducted over the last decades has demonstrated that the first 24 h after symptom onset are critical for the identification of patients who are at risk of developing complications or death. The identification of these subgroups of patients is crucial in order to start an aggressive approach to prevent mortality. In this sense and to avoid unnecessary overtreatment, thereby reducing the financial implications, the proper identification of mild disease is also important and necessary. A large number of multifactorial scoring systems and biochemical markers are described to predict the severity. Despite recent progress in understanding the pathophysiology of AP, more research is needed to enable a faster and more accurate prediction of severe AP. This review provides an overview of the available multifactorial scoring systems and biochemical markers for predicting severe AP with a special focus on their advantages and limitations.

## 1. Introduction

**Figure 1:**
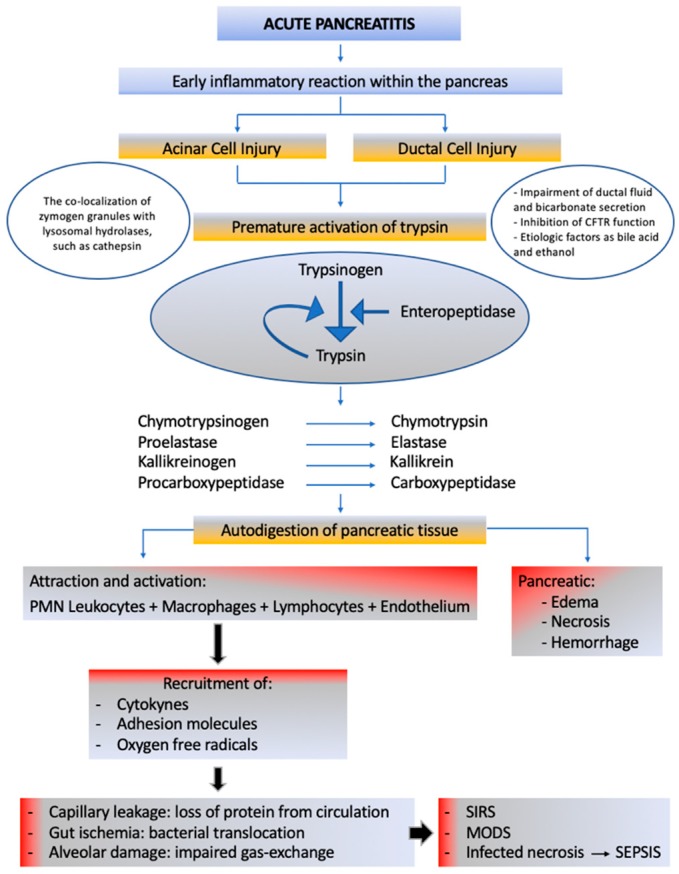
A schematic overview of acute pancreatitis’s pathogenesis. The premature activation of trypsin caused by acinar and ductal cell injury. Attraction and activation of leukocyte, macrophages, lymphocytes, and endothelium causes the release of cytokines, adhesion molecules, and oxygen free radicals. They are responsible for capillary leakage, gut ischemia, and bacterial translocation and alveolar damage. Systemic Inflammatory Response Syndrome (SIRS) is a result of all these events, which may advance to multiorgan disfunction, as well as infection of pancreatic necrosis and sepsis. CFTR: Cystic Fibrosis Transmembrane Conductance Regulator; PMN: polymorphonuclear; SIRS: Systemic Inflammatory Response Syndrome; MODS: Multiple Organ Dysfunction Syndrome [6,10,16,17,18,19,20,21,22,23].

Acute pancreatitis (AP) is a severe inflammation of the pancreas presented with sudden onset and severe abdominal pain with a high morbidity and mortality rate, if accompanied by severe local and systemic complications. It is the most common gastrointestinal cause of hospitalization [1], associated with high financial burdens [2]. Several studies have shown that the incidence of AP is increasing [3,4], probably as a result of a combination of risk factors, such as obesity and gallstone disease [5]. The overall mortality rate is 3% to 10%, but patients with the severe form of the disease are at an increased risk of death, with a mortality rate of 36% to 50% [2,6,7]. Although its etiology is complex and not known for certain, the two most common causes are gallstones and alcohol [3,8]. Numerous studies have been published about the pathogenesis of AP; however, the precise mechanism behind this pathology remains unclear [9]. Even with the proposal of several mechanisms about the pathophysiological process of AP, none are totally enlightening [10]. Some of the hypotheses include acinar and ductal premature activation of trypsin, leukocyte attraction and activation, recruitment of cytokines, adhesion molecules, and oxygen free radicals, which lead to mitochondrial dysfunction and microcirculatory injury [9,11,12,13]. Initial AP events take place in the acinar cells [14]. Acinar cells can act as inflammatory cells as they respond, synthesize, and release cytokines, chemokines, and adhesion molecules [15]. Most research related to the pathophysiology of acute pancreatitis has been directed to acinar cells [16]. However, there is recent evidence that not only the acinar but ductal cell is also involved in the initial events of pancreatic damage and in the development of the inflammatory process [17]. The main function of the pancreatic duct is the bicarbonate and fluid secretion, which can be influenced by alcohol, fatty acids, and bile acids [16,18,19]. The changes in either fluid or bicarbonate secretion are related to changes in cystic fibrosis transmembrane conductance regulator (CFTR) function and expression [17]. It is also important to note that acinar–ductal cell interaction is crucial to the entire process of acute pancreatitis [20]. An excessive inflammatory response is the common aspect of these mechanisms. These can only explain certain aspects of pathogenesis or specific characteristics related to its etiology. The major obstacles in the study of pathogenesis of AP is its rapid course and relative inaccessibility of pancreatic tissue. To overcome this problem, researchers have now taken to animal models to study the molecular aspects of the pathogenesis of AP [2]. Further complicating the issue are the different results obtained from different animals and models exposed to a similar etiology. The premature activation of trypsin is the most consensual theory as the main mechanism in the initiation of the autodigestion of the pancreatic tissue and subsequently on local and systemic inflammatory processes. AP progression is constituted by three phases: Local inflammation, generalized inflammatory response, and multiorgan dysfunction. Figure 1 is an illustration of the schematic overview of AP’s pathogenesis.

Extensive studies conducted over the last decades have demonstrated that the first 24 h after symptom onset are critical for the identification of which patients are at risk of developing complications or death [24,25].

The term biomarker has been defined by the National Institutes of Health as “a characteristic that is objectively measured and evaluated as an indicator of normal biological processes, pathogenic processes, or pharmacological responses to a therapeutic intervention” [26]. The principal roles of biomarkers are diagnosis, prognosis, and individualization of therapy. Early assessment of severity in AP becomes crucial, especially on the day of admission, as this period is considered a window of opportunity for defining interventions to prevent pancreatic necrosis and organ failure. Nevertheless, none of the current clinical scoring systems or biochemical markers play a definitive role, have widespread applicable value, or are consistently accurate [24,27,28]. Therefore, early identification of the development of severe AP remains a great challenge.

According to the 2012 revision of the Atlanta classification, AP develops in two phases [6]. In the early phase, which is usually over by the end of the first week, systemic disturbances are secondary to local pancreatic inflammation. As the disease progresses, generalized inflammation occurs, defined as systemic inflammatory response syndrome (SIRS). If SIRS is persistent, there is an increased risk of organ failure and local complications. The definition of the duration of organ failure is important. If it resolves within 48 h, it is called of “transient organ failure”; if it persists for more than 48 h, it is called “persistent organ failure”. When organ failure affects more than one organ it is called multiple organ failure (MOF) or multiple organ dysfunction syndrome (MODS) [6]. The late phase is characterized by the persistence of systemic signs of inflammation or by local complications. At this stage, the immune system is downregulated, making the (peri) pancreatic tissue more susceptible to infection from intestinal bacterial translocation. The resulting sepsis and multiorgan failure are subsequently the major causes of late morbidity and mortality.

In 65% to 85% of cases, AP is self-limited, not requiring specific treatment other than parenteral intravenous fluid, analgesics, and supportive care [25]. The remaining may suffer from severe attacks, with a high morbidity and mortality. This subgroup of patients need to be identified early in the course of the disease and need to be aggressively treated to prevent mortality [25]. In this sense and in order to avoid unnecessary overtreatment, thereby reducing the financial implications, proper identification of the mild disease is also important and necessary.

Severity assessment in this condition was first started in 1974 by Ranson et al. [29]. Since then other multifactorial scoring systems applying common clinical and biochemical parameters, have been defined to predict the severity. Despite recent progress in understanding the pathophysiology of AP, more research is needed to enable a faster and more accurate prediction of severe AP.

In this review, an overview of the multifactorial scoring systems and biochemical markers for predicting severe AP will be discussed, with a special focus on their advantages and limitations.

## 2. Clinical Assessment

Clinical assessment is an evaluation of a patient’s physical condition and prognosis based on information gathered from their physical condition and the patient’s medical history. The differentiation between mild and severe AP based on clinical assessment has been evaluated in several studies [30,31,32]. Wilson et al. [30] evaluated each patient on admission, at 24 and 48 h after admission, and classified AP as mild, moderate, and severe based on the presence or absence of shock and respiratory distress; the adequacy of the peripheral perfusion and urine output; fever; body wall staining; and the degree of abdominal tenderness, distension, and ileus. They verified that the sensibility, specificity, positive predictive value (PPV), negative predictive value (NPV), and accuracy were, on admission, 34%, 98%, 87%, 83%, and 83%, respectively, and at 24 and 48 h after admission, 47%, 100%, 100%, 86%, and 87%, respectively, in both. They concluded that on admission, the clinical assessment was much less sensitive. Previous studies suggested that at 48 h after admission, clinical assessment was a good tool for predicting the severity of AP [32]. Pagliari et al. [33] in a recent review, highlighted the importance of clinical evaluation not only in diagnosis but also in clinical course.

## 3. Multifactorial Scoring Systems

### 3.1. Ranson Score

The Ranson score was published in 1974 as the first specific multifactorial scoring system for AP [34]. It was primarily designed for patients with acute alcoholic pancreatitis, consisting of 11 parameters identified as significant prognostic factors: Five parameters measured at admission and six during the next 48 h. Ranson et al. [35] in 1979 modified the original score, adapting it for patients with acute biliary pancreatitis. Mortality increases with an increasing score. A score between 1 to 3 criteria represents mild AP; the mortality rate rises significantly with four or more criteria, being 100% in those with six or more [34]. Hagjer et al. [36] evaluated the Ranson score as a predictive tool for AP severity, organ failure, necrosis, and mortality, describing an area under the receiver-operating curve (AUC) of 0.810, 0.839, 0.556, and 0.803, respectively. The disadvantages of the Ranson score is that it requires 48 h to be completed, uses parameters that are not usually evaluated in clinical practice, and is missing a potentially valuable early therapeutic window.

### 3.2. Glasgow Score

Imrie et al. [37] proposed a modification of the Ranson scoring system, where they excluded hematocrit, base deficit, and fluid sequestration, and added albumin and changed the cut-off points. This score was later simplified [38,39]. The Glasgow score is a good prognostic tool for mortality, regardless of the etiology [30]. Buxbaum et al. [40] showed an AUC for the Glasgow score to predict an AP severity of 0.73. In turn, Kiat et al. [41] verified an AUC for a severity of 0.784. The main disadvantage of this score is similar to Ranson’s score, requiring 48 h for a final calculation punctuation.

### 3.3. Acute Physiology and Chronic Health Evaluation II Score

The APACHE score was originally designed to assess the severity of patients with acute illness admitted to intensive care units (ICUs) in the 1970s. In the 1980s, Wagner et al. [42] described a simplification of the APACHE score, since it was the most widely used scoring system for severity assessment, designating it as APACHE-II. The APACHE-II has been used as a reference standard in several studies to evaluate new prognostic scoring systems or to identify individual risk factors for severe outcomes [42]. This score, although widely used in different types of studies is not specific to AP. Wu et al. [43] verified that only 2.2% of included patients with AP had complete data for the APACHE-II classification. Despite this complexity, the APACHE-II score requires 14 parameters. Using the worst data during the initial 24 h after admission, several studies have shown a correlation between a higher APACHE-II score at admission to into the first 72 h, with a higher mortality rate (<4% with an APACHE-II score <8 and 11% to 18% with an APACHE-II score ≥8) [44,45,46]. When the severity of AP is assessed, this score is powerless in distinguishing between interstitial and necrotizing AP, which is associated with a different prognosis [47]. Chatzicostas et al. [48] verified that the APACHE-II score generated within the first 24 h had a PPV of only 43% and NPV of 86% for severe AP. It can be used to assess the severity of the patient on a day-to-day basis. Papachristou et al. [49], recognizing obesity as a risk factor for complications of AP, proposed APACHE-O, an improvement on the APACHE-II accuracy. However, they concluded that APACHE-O did not improve the accuracy of APACHE-II (AUC 0.895 for APACHE-O and 0.893 for APACHE-II). Harshit et al. [50] compared APACHE-II with other scores in predicting the severity of AP and concluded that this score was an effective prognostic scoring system able to predict the severity of AP. The disadvantage of APACHE-II is the need for 24 h for the final determination of AP severity and it is complex and difficult to use in clinical practice.

### 3.4. Bedside Index of Severity in Acute Pancreatitis Score

The Bedside Index for Severity in Acute Pancreatitis (BISAP) was developed in 2008 by Wu et al. [43]. They described it as an easy score that is calculated from data available in the first 24 h after admission [51]. This feature is extremely significant given that the first 24 to 48 h are the most crucial and decisive time window in the management of AP. The performance of the BISAP score in predicting severe AP has been corroborated by numerous studies [43,44,45,52,53,54]. The BISAP score was aimed for use during the first 24 h of admission to hospital and includes five parameters [43,55]. This score was derived using data from a population of 17,992 patients and validated on a population of 18,256 patients in the USA and could predict in-hospital mortality from AP with an AUC of 0.83 (95% CI:0.8–0.85) [56]. The BISAP score registered an identical efficiency for predicting outcomes as the APACHE-II, but it was easier to determine than the APACHE-II score [53,57]. Khanna et al. [58] showed a sensitivity, specificity, PPV, and NVP for severity of AP of 74.2%, 68.3%, 63.4%, and 77.8%, respectively. In their turn, Hagjer et al. [36] for severity, organ failure, and death associated with AP found an AUC to BISAP score of 0.875, 0.906, and 0.740, respectively. They concluded that BISAP predicts severity, organ failure, and death in AP very well. It is as good as APACHE-II but better than the Ranson criteria, contrast tomography severity index (CTSI), c-reactive protein (CRP), hematocrit, and body mass index (BMI). Although easy to perform, its utility in a clinical setting does not appear appealing.

### 3.5. Systemic Inflammatory Response Syndrome

The SIRS score is simple and widely used in the clinical setting. According to Banks et al. [6], during the early phase of AP, local pancreatic injury will provoke systemic disturbances. It is in this phase that cytokine cascades are activated by this local inflammation, which clinically manifest as SIRS. If SIRS (≥2) persists for more than 48 h after admission, there is an increased risk of developing multiorgan dysfunction, determined by the modified Marshall scoring (MMS) system [6]. Although several studies have shown that the SIRS score can predict the severity of AP [59,60], Li et al. [61] showed that SIRS had a medium performance with the lowest AUC when compared with the APACHE-II, Ranson score, BISAP, sequential organ failure assessment (SOFA), and MMS, in predicting severe AP, pancreatic necrosis, and infected pancreatic necrosis (IPN). They concluded that the SIRS score is not a priority in predicting severe AP, pancreatic necrosis, and IPN.

### 3.6. Pancreatitis Activity Scoring System

An international panel of experts developed the acute Pancreatitis Activity Score System (PASS) to measure the disease activity in patients with AP [62]. In this score, five parameters were included: Organ failure, SIRS, abdominal pain, requirement for opiates, and ability to tolerate oral intake. Buxbaum et al. [40] studied the correlation between the PASS score and the severity of AP, finding an AUC of 0.71. They concluded that the PASS score performance was compared to established systems used to predict severe AP. Ke et al. [63] showed that the admission PASS score was strongly associated with IPN, with an AUC of 0.813, which is was to the APACHE-II score of 0.791, BUN of 0.740, and CRP of 0.619.

## 4. Imaging Scoring Systems and Techniques

### 4.1. Contrast-Enhanced Computed Tomography

Due to its availability and imaging characteristics, contrast-enhanced computed tomography (CECT) is an imaging modality widely used for the diagnosis, assessment of severity, and morphological classification of AP [25]. It is well known that CECT imaging helps in the delineation of pancreatic and/or peripancreatic necrosis, inflammatory changes, and the characterization of the morphology of fluid collections, making it an excellent tool for the therapeutic decision and approach and monitoring of treatment response [64]. Despite all these features, the majority of the patients do not require computed tomography (CT) for the diagnosis of AP, and the CECT is not indicated in patients who are clinically stable and with clinical improvement [6,25]. CECT also fails to predict the formation of necrosis when performed very early after the beginning of symptoms. The ideal time for performing this imaging technique is at least after 72 h after\onset [57,64]. The sensitivity and specificity of perfusion CT for predicting necrotizing pancreatitis was given as 87.5% and 100%, respectively [65]. These data suggest that perfusion CT might be an alternative measure to the clinical scores and CECT for risk stratification in severe AP. The severity of AP was assessed using several CT scoring systems. The first score was described by Balthazar et al. [66]. By using early CT signs of AP, they were able to develop a gradient system to predict the severity based on the overall evaluation of the size, contour, and density of (peri)pancreatic abnormalities. In 1990, Balthazar et al. [67] validated the CTSI by combining their original score system with the presence and extension of pancreatic necrosis. Although having better prognostic accuracy than the original score, several limitations were described associated with CTSI, which led to the proposal by Mortelé et al. [68] of a new score, the modified CT scoring system (mCTSI). This score proved to have better accuracy for infection, organ failure, the need for surgical or percutaneous intervention, the length of hospital stays, and death. Raghuwanshi et al. [69] verified that mCTSI was more accurate, easier to calculate, and reduced inter-observer variation compared to CTSI. Avanesov et al. [70] concluded that mCTSI was more accurate in predicting short-term mortality and CTSI for predicting the need of intervention. Despite all the improvements to the first score, CT scanning did not predict the severity of AP better than conventional systems and it is not recommended on admission purely for severity assessment [71,72].

### 4.2. Transabdominal Ultrasonography

Conventional transabdominal ultrasound (US) plays only a limited role in the staging of AP, since the detection of pancreatic necrosis is difficult because this exam cannot assess organ perfusion [73]. Through the use of contrast enhancers, it can provide characterization of pancreatic vascularization behavior and can differentiate between areas of inflammation (hypervascularized) and areas of necrosis (hypovascularized or non-vascularized) [74,75]. Golea et al. [74] concluded that contrast-enhanced ultrasound (CEUS) is usefulness in the quantification of the necrosis area in AP. Cai et al. [76] conducted a study to evaluate the accuracy of conventional US and CEUS in patients with AP, concluding that CEUS is a reliable method for the diagnosis and prognosis of AP, and it may serve as a substitute for CECT. Skouras et al. [77], by studying the characteristics of lung ultrasonography and its role in the diagnosis of respiratory dysfunction, proposed that this exam may be an adjuvant in the assessment of the severity of pancreatitis.

### 4.3. Endoscopic Ultrasonography

Endoscopic ultrasonography (EUS) allows for the visualization of the whole pancreas with details of the parenchymal structure and peripancreatic changes due to its high resolution images [78,79]. EUS can also determine the etiology of idiopathic AP [80], the presence of microlithiasis, occult pancreatic malignancies, morphologic changes as pancreas divisium, and evaluate chronic pancreatitis. The close proximity of the endoscopic ultrasound probe to the pancreas results in high spatial resolution that is superior to that of CT and magnetic resonance imaging (MRI) [78]. Sotoudehmanesh et al. [78] concluded that peripancreatic edema in EUS may be a new imaging criterion for the early prediction of the severity of AP (sensitivity, specificity, and accuracy: 65.8%, 75.5%, and 72.2%, respectively). They verified that the cutoff day for the detection of severe AP is the second day of admission, which is very important for the decision of therapeutic modality. Khanna et al. [58] studied the presence of pancreatic and extrapancreatic necrosis and concluded that patients with acute necrotizing pancreatitis have multiple hypoechoic or hyperechoic areas in the pancreas that were not present in patients with mild AP.

## 5. Metabolic Factors

### 5.1. Metabolic Syndrome

Metabolic syndrome includes hyperglycemia, dyslipidemia, hypertension, and obesity [81]. There has been an increase in its incidence due to lifestyle habits [82]. Few studies related metabolic syndrome with acute pancreatitis and their results are varied. However, a high prevalence of metabolic syndrome has been found in patients with AP and some studies associate this syndrome to severe forms of AP [82,83,84]. Mikolasevic et al. [82] showed that patients with metabolic syndrome had a significantly higher incidence of moderately severe and severe AP in comparison to those without metabolic syndrome. They found that the number of metabolic syndrome components is in relation to the severity of AP. The authors also verified that patients with metabolic syndrome presented with more local and systemic complications.

#### 5.1.1. Increased Body Mass Index

Lankisch et al. [85] studied the role of obesity as a negative prognostic factor of AP. They concluded that increased body weight was associated with increased incidence of early extrapancreatic complications. Several studies have been done between obesity and AP, and they have considered obesity as an independent risk factor for severe AP [86]. Krishna et al. [87] concluded, in their study, that morbid obesity is a negative factor for inpatient hospitalization and it is associated with the mortality, organ failure, and high costs. Dobszain et al. [88], in a meta-analysis, demonstrated that a BMI > 25 increases the risk of severe AP, but not mortality, while a BMI > 30 raises the risk of both severity and mortality of AP. Obesity is a known risk factor for gallstone generation, associated with a high risk for gallstone-related complications and AP complications [89]. Martinez et al. [90] showed a significantly higher rate of severe AP, with an OR of 2.9 (95%CI 1.8–4.6) systemic 2.3 (95% CI 1.4–3.8) and local complications 3.9 (95% CI 2.4–6.6) in obese compared to non-obese patients with AP. Obesity is also related to a poor prognosis of AP, due to the relative increase in the proportion of intrapancreatic fat and the release of high levels of circulating proinflammatory cytokines and adipokines [49,91].

#### 5.1.2. Hyperlipidemia

Hyperlipidemia is the third most common cause of AP [81,92]. Few studies have investigated the relationship between hypertriglyceridemia and the severity of biliary pancreatitis. Valdivielso et al. [92] found that the presence of hypertriglyceridemia was related with severe AP. Zeng et al. [93] verified that hypertriglyceridemia was associated with local and systemic complications. Szentesi et al. [84] verified that hypertriglyceridemia elevated the risk of severe AP.

#### 5.1.3. Hypertension

There are very few studies addressing the relationship between hypertension and AP severity. Szentesi et al. [84] showed that hypertension was independently associated with the severity of AP by increasing the risk of renal failure and prolonged hospitalization, although the underlying mechanism is not known.

### 5.2. Fatty Liver

Fatty liver is commonly associated with pancreaticobiliary diseases, including acute pancreatitis [94]. The incidence of fatty liver as a metabolic condition is increasing considerably. There have been several studies on the association between fatty liver and AP, namely with the severity of AP [81]. Yoon et al. [95], in their study investigating the relationship between fatty liver and the severity of AP, verified that fatty liver may play a prognostic role in this disease and could be incorporated into future predictive scoring models.

### 5.3. Diabetes Mellitus

Some studies have investigated the presence of diabetes mellitus and the severity of AP [81]. However, the results of these studies are contradictory, as some relate the presence of DM to the severity and mortality of PA patients [96,97] while others report that there are no differences between severity and mortality in patients with and without DM [84,98,99].

## 6. Genetic Predisposition

The major challenge of AP is to assess the course of the disease and identify which patients develop mild AP and which patients may have severe AP. This variation in outcome may be related to the genetic polymorphic propensity to produce proinflammatory cytokines [100]. D’Oliveira Martins et al. [101] conducted a study to evaluate the potential modulating role of 15 gene polymorphisms in 10 genes involved in oxidative stress and the apoptotic pathway. This study provided an insight into the potential role of genes polymorphisms in GSTM1, GSTT1, GSTP1, CASP7, CASP8, CASP9, CASP10, LTA, TNFRSF1B, and TP53 gene variants and AP susceptibility.

## 7. Molecular and Serum Markers

### 7.1. Tumor Necrosis Factor-Alpha

Tumor necrosis factor (TNF)-α is an important inflammatory cytokine that participates in the pathogenesis of AP, directly injuring acinar cells and resulting in necrosis, inflammation, and edema [22]. This cytokine, thought to be the first cytokine released, is the main mediator of immune responses [102]. TNF-α expression in the pancreas is increased by the onset of experimental AP. El-Ashmawy et al. [103] conducted a study with a murine model of L-arginine-induced pancreatitis to study the underlying molecular mechanisms of AP. They verified that the pancreatic TNF-α concentration was markedly elevated following L-arginine administration. This may be attributed to the excessive generation of reactive oxygen species (ROS), which activates the nuclear factor kappa B (NF-κB), with subsequent upregulation of various inflammatory cytokines, particularly interleukin (IL)-1β and TNF-α. Levels of TNF-α receptors, indicators of TNF-α activity, have been found to be increased in patients with severe AP, and TNF-α blockade has been shown to reduce mortality and ameliorate the severity in experimental AP [102]. Despite all the knowledge about this cytokine, the results among different studies, regarding its role in predicting the severity in pancreatitis, are conflicting [104]. Paajanen et al. [105] verified that the serum TNF-α levels showed no significant differences between the patients who developed complications and those who did not. Exley et al. [104] concluded that serum TNF-α at presentation, for a cut-off of 35 ng/L, correlated with a worse prognostic score and a severe outcome in all patients (r = 0.36, *p* = 0.027; r = 0.33, *p* < 0.05) and prognostic score, outcome, and mortality in patients with gallstones (r = 0.58, *p* = 0.005; r = 0.60, *p* = 0.005, r = 0.50, *p* = 0.02).

### 7.2. Interleukin-1

IL-1 is well known as an integral early component of the acute inflammatory process [106]. Heresbach et al. [107], in their study to evaluate the severity of AP, found that levels of IL-1 predict severe AP on admission with a similar accuracy to IL-6 (82% versus 88%, respectively) and that IL-1 receptor antagonist had the best accuracy among different markers, including IL-6 and CRP, within the first 48 h. At 48 to 72 h, IL-1 levels have been found to be predictive of pancreatic necrosis with an accuracy of 88%, and the IL-1:IL-1 receptor antagonist ratio could identify septic complications with an accuracy of 72%. Chen et al. [108] evaluated IL-1β on admission and described an accuracy of 82% for a cut-off of ≥1 pg/mL.

### 7.3. Interleukin-6

IL-6 is the principal stimulus for acute-phase protein synthesis in the liver and constitutes the main mediator in the synthesis of fibrinogen, CRP, and hepcidin. The role of IL-6 in the early and accurate prediction of severity in AP was confirmed by numerous studies [21,58,109,110]. Soyalp et al. [111] found that a raised IL-6 level increased in accordance with the severity of pancreatitis, suggesting that IL-6 could act as a prognostic tool of AP. IL-6 has the best sensitivity and specificity for the early assessment of severe AP among the various proinflammatory and anti-inflammatory cytokines. Jiang et al. [112] found a sensitivity and specificity of 100% and 89.7%, respectively, for a cut-off value of 50 pg/mL. Khanna et al. [58] described, for a similar cut-off, a sensitivity of 93.1% and specificity of 96.8%. IL-6 assay has, however, a major drawback, in that its serum concentration decreases very rapidly, as well as its cost and complexity.

### 7.4. Interleukin-8

Among all the cytokines, IL-8 stands out in the AP pathophysiology as it has been demonstrated to be significantly elevated during the development of AP, and the level was reported to be associated with the severity of AP [113]. Several studies have shown promising results in the early prediction of severe AP [114]. Rau et al. [115] verified the role of IL-8 in monitoring major complications in patients of necrotizing pancreatitis with multiorgan failure. Various studies verified that IL-8 levels are increased in the first 24 h after symptom onset, with a rapid decrease after 3 to 5 days being a good marker of multiorgan failure and death from sepsis in patients with AP [116].

### 7.5. C-Reactive Protein

CRP is a positive acute-phase reactant synthesized by the liver, induced by cytokines like IL-6, and its level in the blood increases within hours in response to inflammation and infection [117]. It can be used for diagnosis, prognosis, treatment follow-up, and mortality prediction, especially in inflammatory cases [118]. It takes the CRP level nearly 72 h to peak after the onset of symptoms [58,119]. Mayer et al. [120] performed the first study on the role of CRP in the prediction of the outcome of AP. They concluded that increased levels of CRP may predict the severity of AP. Vasudevan et al. [53] evaluated the early risk assessment of AP by comparing various scores and biochemical markers. Regarding RCP, they found that to predict the severity of AP for a cut-off of ≥82 ng/mL, the odds ratio (OR) was 6.7 (95% CI 1.95–23, *p* = 0.002), and to predict the infected pancreatic necrosis for a cut-off of ≥98 ng/mL, the OR was 2.0 (95% CI 1.06–3.73, *p* = 0.03). They also calculated a predictive value of CRP, with an AUC of 0.8218 for severe AP. It is currently accepted that levels of CRP above 150 mg/dL at 48 h after admission help discriminate severe from mild disease [58], having a sensitivity, specificity, positive predictive value, and negative predictive value of 80%, 75%, 67%, and 86%, respectively, for severe AP [121]. CRP rises steadily in relation to the severity of AP and it is commonly used because it is inexpensive and readily available [117,122,123]. Miko et al. [124] evaluated the severity and mortality related with AP by comparing several score systems and biochemical markers, including CRP. They verified that CRP presented an AUC to the severity of AP of 0.73, with a sensibility and specificity of 71% and 87%, respectively. Farkas et al. [125] developed a multicenter study to assess the role of CRP as a tool to include patients in clinical trials, concluding that although admission has a poor association with mortality and severity of BP, it can be used as an inclusion criterion of patients in clinical trials. CRP is the most promising biochemical marker, with many studies showing a correlation of its high levels with pancreatic necrosis development and a severe AP course [126]. However, CRP levels are influenced by liver disease [127], which may be present in many patients with AP who are obese and/or alcoholics. Despite its high applicability in clinical practice, this inflammatory marker has disadvantages, such as its late peak (48 to 72 h), its nonspeficitity as an inflammatory marker of the pancreas, and its levels are not associated with the infection [128]. Due to their non-specificity, other inflammatory conditions may influence its increase.

### 7.6. Procalcitonin

Procalcitonin (PCT) is a propeptide synthesized by hepatocytes and G-cells of the thyroid gland [58]. It is an acute-phase reactant and several studies have validated its role as an early biochemical marker in infection, sepsis, and multiorgan failure [129]. Severe AP is known to be associated with sepsis, infected pancreatic necrosis, and multiorgan failure, and PCT can be used as an early tool in the prognosis of AP [130,131]. For a faster result, PCT levels can be measured by a semiquantitative strip test with a cut-off level of 0.5 ng/mL [132] while other studies report a better cut-off of 2 ng/mL [133]. An increased PCT level in patients with AP was found to be indicative of severity, pancreatic necrosis, and organ failure. A systematic review found that the sensitivity and specificity of PCT for the development of severe AP were 72% and 86%, respectively, and that the overall AUC was 0.87, for a cut-off value of 0.5 ng/mL [134]. In their study, Khanna et al. [58] found a 100% sensitivity of PCT for predicting organ failure and mortality, and a sensitivity of 86.4% for predicting severe AP. Hagjer et al. [36], in their prospective observational study, concluded that PCT was a promising inflammatory marker with prediction rates similar to the BISAP score. Studies showed that PCT is the most sensitive laboratory test for the detection of pancreatic infection, and low levels appear to be strong negative predictors of infected necrosis [128]. The role of this marker as a tool to identify which patients need antibiotics as well as the duration of the treatment is under study [135]. The major disadvantage of PCT assay is its high cost.

### 7.7. Polymorphonuclear Elastase

PMN elastase is one of the serine proteases found in the granules of neutrophils [136]. Granulocyte infiltration and activation, which occurs as a first-line defense following tissue injury, leads to the release of multiple microbicidal products, including reactive oxygen species, cationic peptides, eicosanoids, and proteolytic enzymes [109,136]. This process also occurs in the early phase of AP [137]. With a cut-off level of 110 µg/L, Dominguez-Muñoz et al. [137] found a sensitivity and specificity of 92% and 91%, respectively, for the detection of severe AP within 48 h of the onset of symptoms. A similar result has been found in several studies [114,117,138].

### 7.8. Tissue Factor

Tissue factor (TF) is a transmembrane glycoprotein involved in the initiation of the coagulation cascade. It is expressed in the vascular adventitia but may also be expressed from leukocytes, endothelial cells, vascular smooth muscle cells, and platelets [139]. Recent studies have shown the efficiency of TF as a marker to assess the severity. Andersson et al. [140] found that TF was superior to CRP for predicting severity. Ou et al. [141] concluded that TF expression and the associated dysfunction of the blood coagulation system are critical factors for the pathogenesis of severe AP. A high serum level early in the course may suggest a role in the pathogenesis of AP and provide a window for therapeutic interventions.

### 7.9. Hepcidin

Hepcidin is a circulating peptide hormone that regulates the entry of iron into plasma. The hepcidin levels increase during inflammation as a result of an increase of IL-6 [142]. Studies have shown that hepcidin is synthesized in the liver, kidney, heart, brain, muscles of the skeleton, and pancreas [143]. Based on this theory, Arabul et al. [144] undertook a single center prospective study to assess its role in predicting the severity in AP. They found that hepcidin is a better predictive marker for severe AP compared to CRP with an AUC of 0.82 versus 0.69, respectively.

### 7.10. Copeptin

Copeptin is a 39 amino acid glycopeptide that is co-synthesized with vasopressin [145]. Its level rises during stress in critically ill patients, making it an independent predictor of survival in this group of patients. Isman et al. [146] verified a significantly high concentration of copeptin on admission in patients with severe AP. They also identified copeptin as a novel prognostic marker to predict local complication, organ failure, and mortality in AP. Nebiker et al. [147] compared copeptin with others markers, including CRP, and concluded that copeptin was associated with disease severity to a similar degree as CRP.

### 7.11. Soluble E-Selectin and Soluble Thrombomodulin

Soluble E-selectin (sES) is an endothelial activation marker, whereas soluble thrombomodulin (sTM) is an endothelial injury marker. During AP, activated neutrophils release elastase, which damages the endothelium. Ida et al. [148] studied these two markers to find their significance in the assessment of severe AP. They concluded that those high levels of soluble ES can be found in all stages of the disease. Soluble ES and TM can be used as a predictive marker of mortality in AP on the first day of admission.

### 7.12. Endothelin I

Elevated levels of endothelin I (ET-1) have been found to be associated with AP, with a strong correlation with disease severity. Milnerowicz et al. [149] measured plasma ET-1 levels, verifying that ET-1 can be used as a marker of both the progression of the disease and monitoring treatment [150]. They also concluded that an increase in levels of ET-1 between the fifth and seventh days of treatment may indicate irreversible ischemia lesions in the pancreas and the development of pancreatic necrosis.

### 7.13. Matrix Metalloproteinase-9

Matrix metalloproteinase (MMP) are a group of enzymes involved in processes, such as inflammation, degradation, and turnover of the extracellular matrix, as well as angiogenesis and tumor growth [151]. The role of MMP-9 has been extensively studied in AP and increased levels of MMP-9 have been found to be of possible prognostic significance [152]. Guo et al. [153] verified a strong association between high levels of MMP-9 and pancreatic necrosis. It can also be used as a marker of disease severity and assessment of the course of the disease [154].

### 7.14. Albumin

Albumin is a negative acute-phase protein synthesized by the liver and its level in the blood decreases during inflammation. Albumin was shown, in previous studies, to be associated with inflammation severity, disease prognosis, and mortality, due to the relationship between inflammation and malnutrition [155]. A few studies have evaluated hypoalbuminemia as a predictor of severe acute pancreatitis [156,157]. Hong et al. [158] concluded that hypoalbuminemia within 24 h of hospital admission is independently associated with increased risk of the development of persistent organ failure and death in AP.

### 7.15. Total Calcium

Low serum ionized calcium (Ca^2+^) levels have been demonstrated as playing an important role in detecting patients with severe AP [159]. Gutiérrez- Jiménez et al. [160] verified a sensitivity, specificity, positive predictive value, and negative predictive value of 67%, 82%, 27%, and 96% at a maximum cut-off of 7.5 mg/dL for total calcium in predicting severe AP. Yu et al. [161] showed that low serum Ca^2+^ is an independent risk factor affecting the severity of AP.

### 7.16. Pancreatic Protease Activation Peptides

Trypsinogen activation peptide (TAP) is a cleavage product of trypsinogen, which is released into systemic circulation with zymogen granule activation. Because of its low molecular weight, TAP is rapidly excreted in urine and is easily detected in both urine and serum. Similarly, carboxypeptidase B activation peptide (CAPAP) is a peptide fragment of procarboxypeptidase B, a large cytosolic protein in acinar cells [162]. Wu et al. [163] developed a study to assess the role of trypsinogen-2 in predicting local pancreatic complications. They concluded that urinary trypsinogen-2 level >500 µg/L independently predicted local complications of AP. On the other hand, Yasuda et al. [164] studied the role of urinary trypsinogen-2 and TAP to assess extra-pancreatic complications. They confirmed that the urinary trypsinogen-2 dipstick test was useful as a marker for the diagnosis of AP. The authors also verified that both trypsinogen-2 and TAP may be useful markers to determine extra-pancreatic inflammation in AP. Deng et al. [165], in their systematic review and meta-analysis, verified that both serum and urinary CAPAP have the potential to act as a stratification marker on admission in predicting the severity of AP.

### 7.17. Red Blood Cell Distribution Width

Red blood distribution width (RDW) is a routine parameter of the complete blood count test, which is easily obtained and inexpensive [166,167]. It is commonly performed in the assessment of almost all patients at the time of admission. Conventionally, RDW has been used as a tool to explore the etiologies of anemia. During the past decade, RDW has been associated with inflammatory parameters, such as CRP, IL-6, and fibrinogen [167]. It has also been associated disease activity and the prognosis of various diseases, such as malignancies, heart failure, autoimmune diseases, and hepatocellular carcinoma [166]. To date, multiple studies have examined the usefulness of RDW in determining the prognosis of AP at the time of admission, but the results have not been consistent [166]. Zhou et al. [168] studied the predicting value of several markers, including RDW. They concluded that RDW is a convenient and reliable indicator for the prediction of not only severe AP but also mortality.

### 7.18. Blood Urea Nitrogen

Several prognostic scoring systems, including the Ranson score, incorporate blood urea nitrogen for predicting the severity and mortality of AP [43,169]. This marker provides information on changes in intravascular volume status. Therefore, it could be used in monitoring early responses to initial fluid resuscitation [170]. Wu et al. verified, in a large prospective multicentric study, that blood urea nitrogen (BUN) was an accurate predictor of mortality for a cut-off of BUN ≥20 md/dL on admission, where the associated OR was 4.6 for mortality [56]. They also concluded that a rise in the levels of BUN within the first two days has been correlated with increased mortality.

### 7.19. Hematocrit

Hemoconcentration may be a marker that translates pancreatic microcirculation insufficiency responsible for the development of necrosis [171]. Hemoconcentration on admission, as defined by initial hematocrit, has been described as a useful tool of prognosis of AP. Early hemoconcentration has been shown to be associated with an increased risk of both necrosis and severe AP [172]. Elevated levels of hematocrit on admission (hematocrit >44%) have been associated with the development of pancreatic necrosis, organ failure, as well as prolonged hospitalization and need for intensive care [173,174,175].

### 7.20. Creatinine

Renal involvement has frequently been reported in the course of AP [176]. Muddana et al. [177], in their study for a cut-off of creatinine >1.8 mg/dL within 48 h after hospital admission, showed an OR of 34.5 (CI 95% 7.3 to 169) for the development of pancreatic necrosis when compared with admission hematocrit and BUN levels. They concluded that an increase in creatinine concentration within 48 h of admission is strongly associated with the development of pancreatic necrosis.

### 7.21. Proteinuria

Increased renal permeability is verified after burn injury, trauma, ischemia, and surgery conditioning low proteinuria [178]. The degree of proteinuria correlates with severity and outcome in a variety of pathologies [178,179]. This marker can be detected by a urine dipstick that allows for easy and inexpensive results. Shearmen et al. evaluated the levels of urine excretion of albumin and IgG in patients with AP. They verified that during the first 36 h, levels were significantly higher in patients with severe AP, concluding that low proteinuria may be reflected the severity of inflammation in AP. However, Zuidema et al. [179], in their study that compared the relation between proteinuria and severity of AP, infection complications, need for invasive approach, intensive care stay, and in-hospital mortality, concluded that proteinuria was inferior to the CRP. Despite the differences verified between the two studies, future research with a larger sample population may contribute to an evaluation of the role of proteinuria as a prognostic marker of pancreatitis.

### 7.22. Angiopoietin 2

Angiopoietins are a novel class of angiogenic growth factors that act selectively on endothelial cells [180]. Angiopoietin-1 and angiopoietin-2 are modulators of vascular permeability. Angiopoietin-2 has been recently evaluated as a marker of persistent organ failure in patients with severe AP, since it is an endothelium-specific growth factor regulated by proinflammatory stimuli. This results in destabilization of the vascular endothelium and an increase of vascular leakage [180]. Whitcomb et al. [181], in a multicenter prospective study, assessed the role of angiopoietin-2 as an early marker of persistent organ failure in patients of severe AP. They found that angiopoietin-2 levels on the day of admission were significantly higher in patients with persistent organ failure, with a sensitivity, specificity, and area under the curve of 90%, 67%, and 0.81, respectively.

### 7.23. Oleic Acid Chlorohydrin

From studies of the pathophysiology of AP in murine models, it was found that infiltration and activation of pancreatic polymorphonuclear neutrophils and in the surrounding areas of adipose tissue results in the generation of hypochlorous acid and fatty acid chlorohydrins (FA-CI), with oleic acid chlorohydrin (OAC) being the most abundant. Franco-Pons et al. [182] evaluated the generation of halogenated fatty acids in the areas of fat necrosis in rats and they concluded that during AP, adipose tissue release FA-CI, which exacerbated the SIRS. Based on these results, de-Madaria et al. [183] evaluated the role of OAC in a prospective and multicenter cohort study as marker of the severity of AP. They concluded that OAC is generated during AP, its levels can be measured in plasma, and these correlate with AP severity.

### 7.24. D-Dimer

The activation of the coagulation cascade has been known to occur during the early phase of AP, and AP induces the formation of venous thrombosis [184]. Thrombosis is a vascular event associated with AP complications that cause morbidity and mortality [185]. D-dimer can be used as a potential severity marker in AP. Significantly different levels of D-dimer have been identified in patients with mild or severe AP. Radenkovic et al. [186] verified D-dimer as a novel marker for predicting organ failure, with a sensitivity of 90% and NPV of 96% for a cut-off level of 414.00 microg/L. Several studies show that D-dimer is an easy, useful, and inexpensive early prognostic marker of severe AP [186,187].

### 7.25. Histones

Histones have been examined in experimental AP murine models, and most have shown a correlation between circulating levels and AP severity [188,189]. Histones are essential for DNA packaging and genetic regulation. In cases of severe sepsis, such as severe AP, circulating histones are detectable in the blood [190]. Kang et al. [191] suggested that circulating histones behave as damage-associated molecular pattern molecules that cause inflammation and contribute to SIRS and death. Histones stimulate cytokine release. Ou et al. [188] in AP murine models found that circulating histones increased significantly in acute necrotizing pancreatitis due to extensive pancreatic acinar cell death. Liu et al. [189] studied a total of 236 consecutive patients with AP and concluded that assessing circulating histones in plasma within 48 h of symptom onset can predict persistent organ failure and mortality. Although these two studies are encouraging for the use of circulating histones in predicting AP severity, Biberci Keskin et al. [192] concluded, in a small patient sample, that serum histone levels did not significantly differ between the severe and mild AP groups.

### 7.26. Inter-Cellular Adhesion Molecule 1

Inter-cellular adhesion molecule 1 (ICAM-1) can play an important role in many biological processes, such as inflammation, by the adhesion of cell-to-cell or cell-to-extracellular matrix [193]. Zhu et al. [194], in their prospective study population of 86 consecutive patients with AP, they verified that for a cut-off of 25 ng/mL ICAM-1 was a good marker for distinguishing mild from severe AP, with a sensitivity and specificity of 61.1% and 71.4%, respectively. They also concluded that the ICAM-1 test was a simple, rapid, and reliable method in clinical practice.

## 8. Proteomic Profiling

Proteomics or protein pattern analysis is the characterization and quantitation of proteins in tissue and body fluids constituting a novel and rapidly expanding field used to compare protein expression patterns between disease states [195,196]. Fétaud et al. [197] concluded that proteomic analysis is a very interesting tool to identify changes characterizing pancreatic tissue damage and new potential biomarkers of AP severity. This method may increase our knowledge of the molecular mechanisms underlying AP and thus enhance new diagnostic and prognostic biomarkers [198,199]. The serum proteomic profile has features that can differentiate mild from severe AP. Papachristou et al. [196] verified 18 different signal intensity clusters out of 72 spectral clusters. Classification and regression tree (CART) analysis showed a primary splitter at 11,720 Da. After analysis, it was found to have a sensitivity of 100% and specificity of 81% in discriminating mild from severe acute pancreatitis. Proteomic profiling has also been used to differentiate disease states from non-disease states. In this respect, it could be used to assess disease severity and predict the clinical course of AP. A recent study assessed the use of proteomic profiling in discriminating severe from mild AP early in the course of the disease. The initial analysis of admission serum from 28 AP patients (7 severe and 21 mild) provided specific distinctive proteomic patterns, including peak clusters of interest relating to serum amyloid A.

## 9. Metabolomic Profiling

Metabolomics is a systematic approach for the analysis of biological samples. It can provide detailed information of the metabolic changes taking place in an organism. Metabolite profiling by using nuclear magnetic resonance spectroscopy and mass spectrometry has been widely applied for analyzing physiological and/or pathological conditions, such as AP. In the presence of pancreatic inflammation, metabolic abnormalities appear before both the transformation of tissue structure and changes in function [200]. The identification of these metabolic changes may promote an understanding of the pathophysiological events in AP. Ma et al. [200] verified a correlation between glucose, lactate, betaine, choline, glycerophosphocholine/phosphocoline, leucine/iso-leucine/valine, and several lipids with acute necrotizing pancreatitis. Xiao et al. [201] showed that 3-hydroxybutyric acid and citric acid were potential biomarkers of the prognosis of AP, allowing for the distinction of mild from severe AP.

## 10. Clinical Relevance and Future Directions

Several multifactorial scoring systems and biochemical markers have been evaluated during the last decades.

Numerous biochemical markers have been studied as potential early predictors of the severity of AP so that the therapeutic approach can be optimally adapted to prevent both local and systemic complications. In Table 1 and Table 2, the multifactorial scoring systems and biochemical markers are summarized, which are more studied and representative in the prognosis of AP.

At this moment, no laboratory test has consistent accuracy for the prediction of AP severity [25].

The majority of physicians and guidelines consider the CRP at 48 h after symptom onset as the gold standard for disease severity assessment.

The clinical presentation of AP is very variable. Banks et al. [6] classified AP as mild (uneventful clinical course), moderately severe (local complication or transient organ failure), and severe, characterized by the persistence of multiorgan failure. Patients with severe AP are at an increased risk of developing infected necrosis that is associated with very high morbidity and mortality. The actual management of AP is based on parenteral intravenous fluid therapy, pain control, and adequate nutrition. In cases of infected necrotizing pancreatitis, an endoscopic or surgical step-up approach is evaluated according to the local complication present [202]. Due to its complexity, AP management requires a multidisciplinary approach, such as a surgeon, a gastroenterologist, and a radiologist.

The evolution of new techniques, namely the recognition of both genetic, transcriptomic, proteomic, and metabolomic profiles and functional images, allows for the identification of specific patterns of various pathological processes. These specific patterns can also be used in AP for the selection and validation of new biochemical markers of severity.

## 11. Conclusions

Despite intense research on the pathophysiology of AP, overall disease mortality has not significantly improved. Several studies have shown that early aggressive management reduces morbidity and mortality. In this sense, early diagnosis and timely assessment of the severity are essential. However, an ideal multifactorial scoring system and/or biochemical marker for early assessment of the severity of AP has yet to be defined.

Based on the analysis of available data and evidence, the authors suggest the use of the BISAP score as a multifactorial scoring system and the CRP at 48 h of presentation as the biochemical marker due to their availability, simplicity, and capability to predict AP severity.

It is critical to design and conduct large population-based multicenter studies to identify parameters that allow for the definition of multifactorial scores or biomarkers to predict AP severity and monitor disease progression that can be used routinely.

## Figures and Tables

**Table 1:** Clinically relevant multifactorial scoring systems predicting disease severity in AP.

Multifactorial Scoring System	Cut-Off	Time	AUC ^1^	References
Ranson score	≥3	48 h	0.81–0.88	[36,58,61,124]
Glasgow score	2	48 h	0.73–0.784	[40,41,58]
APACHE-II ^2^	7	24 h	0.80–0.895	[49,50,58,124]
APACHE-O ^3^	7	24 h	0.893	[49]
BISAP ^4^	≥3	24 h	0.79–0.875	[36,43,58,61,124]
SIRS ^5^	≥2	24 h	0.73	[58,61]
PASS ^6^	>160	24 h	0.71	[40]

^1^ AUC: Area under the curve; ^2^ APACHE: Acute Physiology and Chronic Health Evaluation II score; ^3^ APACHE-O: Acute Physiology and Chronic Health Evaluation II score-Obesity; ^4^ BISAP: Bedside Index of Severity in AP score; ^5^ SIRS: Systemic Inflammatory Response Syndrome; ^6^ PASS: Pancreatitis Activity Scoring System.

**Table 2:** Clinically relevant biochemical markers predicting disease severity in AP.

Marker	Cut-Off	Time	AUC ^1^	References
IL ^2^-6	50 pg/mL	24 h	0.90	[58]
IL ^2^-8	196 pg/mL	preoperative	0.778	[115]
CRP ^3^	150 mg/L	24 h	0.61	[119]
CRP ^3^	150 mg/L	48 h	0.73–0.91	[58,119,124]
PCT ^4^	0.5 ng/mL	24 h	0.86–0.91	[131,134]
PCT ^4^	1.77 ng/mL	24 h	0.797	[130]
Hepcidin	234.4 ng/mL	24 h	0.82	[144]
OAC ^5^	32.40 nM	24 h	1	[183]
RDW ^6^	13.35%	24 h	0.787	[168]
BUN ^7^	5.945 mg/dL	24 h	0.677	[168]

^1^ AUC: Area under the curve; ^2^ IL: Interleukin; ^3^ CRP: C-reactive protein; ^4^ PCT: Procalcitonin; ^5^ OAC: Oleic acid chlorohydrin; ^6^ RDW: Red blood cell distribution width; ^7^ BUN: Blood urea nitrogen.

## References

[1] Peery A.F., Dellon E.S., Lund J., Crockett S.D., McGowan C.E., Bulsiewicz W.J., Gangarosa L.M., Thiny M.T., Stizenberg K., Morgan D.R. (2012). Burden of gastrointestinal disease in the United States: 2012 update. Gastroenterology.

[2] Silva-Vaz P., Abrantes A.M., Castelo-Branco M., Gouveia A., Botelho M.F., Tralhão J.G. (2019). Murine Models of Acute Pancreatitis: A Critical Appraisal of Clinical Relevance. Int. J. Mol. Sci..

[3] Samanta J., Dhaka N., Gupta P., Singh A.K., Yadav T.D., Gupta V., Sinha S.K., Kochhar R. (2019). Comparative study of the outcome between alcohol and gallstone pancreatitis in a high-volume tertiary care center. JGH Open Open Access J. Gastroenterol. Hepatol..

[4] Szentesi A., Toth E., Balint E., Fanczal J., Madacsy T., Laczko D., Ignath I., Balazs A., Pallagi P., Maleth J. (2016). Analysis of Research Activity in Gastroenterology: Pancreatitis Is in Real Danger. PLoS ONE.

[5] Yadav D., Ng B., Saul M., Kennard E.D. (2011). Relationship of serum pancreatic enzyme testing trends with the diagnosis of acute pancreatitis. Pancreas.

[6] Banks P.A., Bollen T.L., Dervenis C., Gooszen H.G., Johnson C.D., Sarr M.G., Tsiotos G.G., Vege S.S. (2013). Classification of acute pancreatitis—2012: Revision of the Atlanta classification and definitions by international consensus. Gut.

[7] Parniczky A., Kui B., Szentesi A., Balazs A., Szucs A., Mosztbacher D., Czimmer J., Sarlos P., Bajor J., Godi S. (2016). Prospective, Multicentre, Nationwide Clinical Data from 600 Cases of Acute Pancreatitis. PLoS ONE.

[8] Gurusamy K.S., Nagendran M., Davidson B.R. (2013). Early versus delayed laparoscopic cholecystectomy for acute gallstone pancreatitis. Cochrane Database Syst. Rev..

[9] Saluja A., Dudeja V., Dawra R., Sah R.P. (2019). Early Intra-Acinar Events in Pathogenesis of Pancreatitis. Gastroenterology.

[10] Wang G.J., Gao C.F., Wei D., Wang C., Ding S.Q. (2009). Acute pancreatitis: Etiology and common pathogenesis. World J. Gastroenterol..

[11] Testoni P.A. (2014). Acute recurrent pancreatitis: Etiopathogenesis, diagnosis and treatment. World J. Gastroenterol..

[12] Zhang B., Li S.L., Xie H.L., Fan J.W., Gu C.W., Kang C., Teng M.J. (2018). Effects of silencing the DUSP1 gene using lentiviral vector-mediated siRNA on the release of proinflammatory cytokines through regulation of the MAPK signaling pathway in mice with acute pancreatitis. Int. J. Mol. Med..

[13] Yu J.H., Kim H. (2014). Oxidative stress and inflammatory signaling in cerulein pancreatitis. World J. Gastroenterol..

[14] Criddle D.N. (2016). Reactive oxygen species, Ca^2+^ stores and acute pancreatitis; a step closer to therapy?. Cell Calcium.

[15] Escobar J., Pereda J., Arduini A., Sandoval J., Moreno M.L., Pérez S., Sabater L., Aparisi L., Cassinello N., Hidalgo J. (2012). Oxidative and nitrosative stress in acute pancreatitis. Modulation by pentoxifylline and oxypurinol. Biochem. Pharmacol..

[16] Venglovecz V., Rakonczay Z., Ozsvari B., Takacs T., Lonovics J., Varro A., Gray M.A., Argent B.E., Hegyi P. (2008). Effects of bile acids on pancreatic ductal bicarbonate secretion in guinea pig. Gut.

[17] Maleth J., Balazs A., Pallagi P., Balla Z., Kui B., Katona M., Judak L., Nemeth I., Kemeny L.V., Rakonczay Z. (2015). Alcohol disrupts levels and function of the cystic fibrosis transmembrane conductance regulator to promote development of pancreatitis. Gastroenterology.

[18] Lee M.G., Muallem S. (2008). Pancreatitis: The neglected duct. Gut.

[19] Toth E., Maleth J., Zavogyan N., Fanczal J., Grassalkovich A., Erdos R., Pallagi P., Horvath G., Tretter L., Balint E.R. (2019). Novel mitochondrial transition pore inhibitor N-methyl-4-isoleucine cyclosporin is a new therapeutic option in acute pancreatitis. J. Physiol..

[20] Pallagi P., Venglovecz V., Rakonczay Z., Borka K., Korompay A., Ozsvari B., Judak L., Sahin-Toth M., Geisz A., Schnur A. (2011). Trypsin reduces pancreatic ductal bicarbonate secretion by inhibiting CFTR Cl(-) channels and luminal anion exchangers. Gastroenterology.

[21] Rau B.M., Kruger C.M., Schilling M.K. (2005). Anti-cytokine strategies in acute pancreatitis: Pathophysiological insights and clinical implications. Rocz. Akad. Med. Bialymst. (1995).

[22] Chen P., Huang L., Sun Y., Yuan Y. (2011). Upregulation of PIAS1 protects against sodium taurocholate-induced severe acute pancreatitis associated with acute lung injury. Cytokine.

[23] Steer M.L., Meldolesi J., Figarella C. (1984). Pancreatitis. The role of lysosomes. Dig. Dis. Sci..

[24] Zhang X.-X., Deng L.-H., Chen W.-W., Shi N., Jin T., Lin Z.-Q., Ma Y., Jiang K., Yang X.-N., Xia Q. (2017). Circulating microRNA 216 as a marker for the early identification of severe acute pancreatitis. Am. J. Med. Sci..

[25] Leppaniemi A., Tolonen M., Tarasconi A., Segovia-Lohse H., Gamberini E., Kirkpatrick A.W., Ball C.G., Parry N., Sartelli M., Wolbrink D. (2019). 2019 WSES guidelines for the management of severe acute pancreatitis. World J. Emerg. Surg..

[26] Strimbu K., Tavel J.A. (2010). What are biomarkers?. Curr. Opin. HIV AIDS.

[27] Kaplan M., Ates I., Akpinar M.Y., Yuksel M., Kuzu U.B., Kacar S., Coskun O., Kayacetin E. (2017). Predictive value of C-reactive protein/albumin ratio in acute pancreatitis. Hepatobiliary Pancreat. Dis. Int..

[28] Tenner S., Baillie J., DeWitt J., Vege S.S. (2013). American College of Gastroenterology guideline: Management of acute pancreatitis. Am. J. Gastroenterol..

[29] Ranson J., Rifkind K., Turner J. (1976). Prognostic signs and nonoperative peritoneal lavage in acute pancreatitis. Surg. Gynecol. Obstet..

[30] Wilson C., Heath D.I., Imrie C.W. (1990). Prediction of outcome in acute pancreatitis: A comparative study of APACHE II, clinical assessment and multiple factor scoring systems. Br. J. Surg..

[31] Corfield A.P., Cooper M.J., Williamson R.C., Mayer A.D., McMahon M.J., Dickson A.P., Shearer M.G., Imrie C.W. (1985). Prediction of severity in acute pancreatitis: Prospective comparison of three prognostic indices. Lancet.

[32] McMahon M.J., Playforth M.J., Pickford I.R. (1980). A compaative study of methods for the prediction of severity of attacks of acute pancreatitis. Br. J. Surg..

[33] Pagliari D., Brizi M.G., Saviano A., Mancarella F.A., Dal Lago A.A., Serricchio M.L., Newton E.E., Attili F., Manfredi R., Gasbarrini A. (2019). Clinical assessment and management of severe acute pancreatitis: A multi-disciplinary approach in the XXI century. Eur. Rev. Med Pharmacol. Sci..

[34] Ranson J.H., Rifkind K.M., Roses D.F., Fink S.D., Eng K., Spencer F.C. (1974). Prognostic signs and the role of operative management in acute pancreatitis. Surg. Gynecol. Obstet..

[35] Ranson J.H. (1979). The timing of biliary surgery in acute pancreatitis. Ann. Surg..

[36] Hagjer S., Kumar N. (2018). Evaluation of the BISAP scoring system in prognostication of acute pancreatitis—A prospective observational study. Int. J. Surg..

[37] Imrie C.W., Benjamin I.S., Ferguson J.C., McKay A.J., Mackenzie I., O’Neill J., Blumgart L.H. (1978). A single-centre double-blind trial of Trasylol therapy in primary acute pancreatitis. Br. J. Surg..

[38] Osborne D.H., Imrie C.W., Carter D.C. (1981). Biliary surgery in the same admission for gallstone-associated acute pancreatitis. Br. J. Surg..

[39] Blamey S.L., Imrie C.W., O’Neill J., Gilmour W.H., Carter D.C. (1984). Prognostic factors in acute pancreatitis. Gut.

[40] Buxbaum J., Quezada M., Chong B., Gupta N., Yu C.Y., Lane C., Da B., Leung K., Shulman I., Pandol S. (2018). The Pancreatitis Activity Scoring System predicts clinical outcomes in acute pancreatitis: Findings from a prospective cohort study. Am. J. Gastroenterol..

[41] Kiat T.T.J., Gunasekaran S.K., Junnarkar S.P., Low J.K., Woon W., Shelat V.G. (2018). Are traditional scoring systems for severity stratification of acute pancreatitis sufficient?. Ann. Hepato-Biliary-Pancreat. Surg..

[42] Knaus W.A., Draper E.A., Wagner D.P., Zimmerman J.E. (1985). APACHE II: A severity of disease classification system. Crit. Care Med..

[43] Wu B.U., Johannes R.S., Sun X., Tabak Y., Conwell D.L., Banks P.A. (2008). The early prediction of mortality in acute pancreatitis: A large population-based study. Gut.

[44] Malangoni M.A., Martin A.S. (2005). Outcome of severe acute pancreatitis. Am. J. Surg..

[45] Blum T., Maisonneuve P., Lowenfels A.B., Lankisch P.G. (2001). Fatal outcome in acute pancreatitis: Its occurrence and early prediction. Pancreatology.

[46] Johnson C., Abu-Hilal M. (2004). Persistent organ failure during the first week as a marker of fatal outcome in acute pancreatitis. Gut.

[47] Perez A., Whang E.E., Brooks D.C., Moore F.D., Hughes M.D., Sica G.T., Zinner M.J., Ashley S.W., Banks P.A. (2002). Is severity of necrotizing pancreatitis increased in extended necrosis and infected necrosis?. Pancreas.

[48] Chatzicostas C., Roussomoustakaki M., Vlachonikolis I.G., Notas G., Mouzas I., Samonakis D., Kouroumalis E.A. (2002). Comparison of Ranson, APACHE II and APACHE III scoring systems in acute pancreatitis. Pancreas.

[49] Papachristou G.I., Papachristou D.J., Avula H., Slivka A., Whitcomb D.C. (2006). Obesity increases the severity of acute pancreatitis: Performance of APACHE-O score and correlation with the inflammatory response. Pancreatology.

[50] Harshit Kumar A., Singh Griwan M. (2018). A comparison of APACHE II, BISAP, Ranson’s score and modified CTSI in predicting the severity of acute pancreatitis based on the 2012 revised Atlanta Classification. Gastroenterol. Rep..

[51] Chandra S., Murali A., Bansal R., Agarwal D., Holm A. (2017). The Bedside Index for Severity in Acute Pancreatitis: A systematic review of prospective studies to determine predictive performance. J. Community Hosp. Intern. Med. Perspect..

[52] Zheng L., Hong W., Geng W., Stock S., Pan J. (2019). A comparison of the BISAP score and Amylase and BMI (CAB) score versus for predicting severe acute pancreatitis. Acta Gastro-Enterol. Belg..

[53] Vasudevan S., Goswami P., Sonika U., Thakur B., Sreenivas V., Saraya A. (2018). Comparison of Various Scoring Systems and Biochemical Markers in Predicting the Outcome in Acute Pancreatitis. Pancreas.

[54] Arif A., Jaleel F., Rashid K. (2019). Accuracy of BISAP score in prediction of severe acute pancreatitis. Pak. J. Med. Sci..

[55] Park J.Y., Jeon T.J., Ha T.H., Hwang J.T., Sinn D.H., Oh T.H., Shin W.C., Choi W.C. (2013). Bedside index for severity in acute pancreatitis: Comparison with other scoring systems in predicting severity and organ failure. Hepatobiliary Pancreat. Dis. Int..

[56] Wu B.U., Bakker O.J., Papachristou G.I., Besselink M.G., Repas K., van Santvoort H.C., Muddana V., Singh V.K., Whitcomb D.C., Gooszen H.G. (2011). Blood urea nitrogen in the early assessment of acute pancreatitis: An international validation study. Arch. Intern. Med..

[57] Mounzer R., Langmead C.J., Wu B.U., Evans A.C., Bishehsari F., Muddana V., Singh V.K., Slivka A., Whitcomb D.C., Yadav D. (2012). Comparison of existing clinical scoring systems to predict persistent organ failure in patients with acute pancreatitis. Gastroenterology.

[58] Khanna A.K., Meher S., Prakash S., Tiwary S.K., Singh U., Srivastava A., Dixit V. (2013). Comparison of Ranson, Glasgow, MOSS, SIRS, BISAP, APACHE-II, CTSI Scores, IL-6, CRP, and procalcitonin in predicting severity, organ failure, pancreatic necrosis, and mortality in acute pancreatitis. Hpb Surg..

[59] Mofidi R., Duff M., Wigmore S., Madhavan K., Garden O., Parks R. (2006). Association between early systemic inflammatory response, severity of multiorgan dysfunction and death in acute pancreatitis. Br. J. Surg. Inc. Eur. J. Surg. Swiss Surg..

[60] Singh V.K., Wu B.U., Bollen T.L., Repas K., Maurer R., Mortele K.J., Banks P.A. (2009). Early systemic inflammatory response syndrome is associated with severe acute pancreatitis. Clin. Gastroenterol. Hepatol. Off. Clin. Pract. J. Am. Gastroenterol. Assoc..

[61] Li M., Xing X.K., Lu Z.H., Guo F., Su W., Lin Y.J., Wang D.H. (2019). Comparison of Scoring Systems in Predicting Severity and Prognosis of Hypertriglyceridemia-Induced Acute Pancreatitis. Dig. Dis. Sci..

[62] Wu B.U., Batech M., Quezada M., Lew D., Fujikawa K., Kung J., Jamil L.H., Chen W., Afghani E., Reicher S. (2017). Dynamic Measurement of Disease Activity in Acute Pancreatitis: The Pancreatitis Activity Scoring System. Am. J. Gastroenterol..

[63] Ke L., Mao W., Li X., Zhou J., Li G., Ye B., Tong Z., Li W. (2018). The Pancreatitis Activity Scoring System in Predicting Infection of Pancreatic Necrosis. Am. J. Gastroenterol..

[64] Sahu B., Abbey P., Anand R., Kumar A., Tomer S., Malik E. (2017). Severity assessment of acute pancreatitis using CT severity index and modified CT severity index: Correlation with clinical outcomes and severity grading as per the Revised Atlanta Classification. Indian J. Radiol. Imaging.

[65] Yadav A.K., Sharma R., Kandasamy D., Bhalla A.S., Gamanagatti S., Srivastava D.N., Upadhyay A.D., Garg P.K. (2015). Perfusion CT: Can it predict the development of pancreatic necrosis in early stage of severe acute pancreatitis?. Abdom. Imaging.

[66] Balthazar E.J., Ranson J.H., Naidich D.P., Megibow A.J., Caccavale R., Cooper M.M. (1985). Acute pancreatitis: Prognostic value of CT. Radiology.

[67] Balthazar E.J., Robinson D.L., Megibow A.J., Ranson J.H. (1990). Acute pancreatitis: Value of CT in establishing prognosis. Radiology.

[68] Mortele K.J., Mergo P.J., Taylor H.M., Wiesner W., Cantisani V., Ernst M.D., Kalantari B.N., Ros P.R. (2004). Peripancreatic vascular abnormalities complicating acute pancreatitis: Contrast-enhanced helical CT findings. Eur. J. Radiol..

[69] Raghuwanshi S., Gupta R., Vyas M.M., Sharma R. (2016). CT Evaluation of Acute Pancreatitis and its Prognostic Correlation with CT Severity Index. J. Clin. Diagn. Res..

[70] Avanesov M., Loser A., Smagarynska A., Keller S., Guerreiro H., Tahir E., Karul M., Adam G., Yamamura J. (2018). Clinico-radiological comparison and short-term prognosis of single acute pancreatitis and recurrent acute pancreatitis including pancreatic volumetry. PLoS ONE.

[71] London N.J., Neoptolemos J.P., Lavelle J., Bailey I., James D. (1989). Contrast-enhanced abdominal computed tomography scanning and prediction of severity of acute pancreatitis: A prospective study. Br. J. Surg..

[72] Bollen T.L., Singh V.K., Maurer R., Repas K., van Es H.W., Banks P.A., Mortele K.J. (2012). A comparative evaluation of radiologic and clinical scoring systems in the early prediction of severity in acute pancreatitis. Am. J. Gastroenterol..

[73] Rickes S., Monkemuller K., Malfertheiner P. (2007). Acute severe pancreatitis: Contrast-enhanced sonography. Abdom. Imaging.

[74] Golea A., Badea R., Socaciu M., Diaconu B., Iacob D. (2010). Quantitative analysis of tissue perfusion using contrast-enhanced transabdominal ultrasound (CEUS) in the evaluation of the severity of acute pancreatitis. Med. Ultrason..

[75] Rickes S., Uhle C., Kahl S., Kolfenbach S., Monkemuller K., Effenberger O., Malfertheiner P. (2006). Echo enhanced ultrasound: A new valid initial imaging approach for severe acute pancreatitis. Gut.

[76] Cai D., Parajuly S.S., Wang H., Wang X., Ling W., Song B., Li Y., Luo Y. (2016). Accuracy of contrast-enhanced ultrasound compared with conventional ultrasound in acute pancreatitis: Diagnosis and complication monitoring. Exp. Ther. Med..

[77] Skouras C., Davis Z.A., Sharkey J., Parks R.W., Garden O.J., Murchison J.T., Mole D.J. (2016). Lung ultrasonography as a direct measure of evolving respiratory dysfunction and disease severity in patients with acute pancreatitis. HPB Off. J. Int. Hepato Pancreato Biliary Assoc..

[78] Sotoudehmanesh R., Hooshyar A., Kolahdoozan S., Zeinali F., Shahraeeni S., Keshtkar A.A. (2010). Prognostic value of endoscopic ultrasound in acute pancreatitis. Pancreatology.

[79] Rana S.S., Bhasin D.K., Sharma V., Sharma R., Chaudhary V., Chhabra P. (2014). Can early endoscopic ultrasound predict pancreatic necrosis in acute pancreatitis?. Ann. Gastroenterol..

[80] Lopes C.V., Pereira-Lima J., Hartmann A.A. (2019). The role of linear endosonography for the diagnosis of acute pancreatitis when other methods failed. Clin. Res. Hepatol. Gastroenterol..

[81] Li X., Guo X., Ji H., Niu J., Gao P. (2019). Relationships between Metabolic Comorbidities and Occurrence, Severity, and Outcomes in Patients with Acute Pancreatitis: A Narrative Review. BioMed Res. Int..

[82] Mikolasevic I., Milic S., Orlic L., Poropat G., Jakopcic I., Franjic N., Klanac A., Kristo N., Stimac D. (2016). Metabolic syndrome and acute pancreatitis. Eur. J. Intern. Med..

[83] Deng L.H., Xue P., Xia Q., Yang X.N., Wan M.H. (2008). Effect of admission hypertriglyceridemia on the episodes of severe acute pancreatitis. World J. Gastroenterol..

[84] Szentesi A., Parniczky A., Vincze A., Bajor J., Godi S., Sarlos P., Gede N., Izbeki F., Halasz A., Marta K. (2019). Multiple Hits in Acute Pancreatitis: Components of Metabolic Syndrome Synergize Each Other’s Deteriorating Effects. Front. Physiol..

[85] Lankisch P.G., Schirren C.A. (1990). Increased body weight as a prognostic parameter for complications in the course of acute pancreatitis. Pancreas.

[86] Ye C., Liu L., Ma X., Tong H., Gao J., Tai Y., Huang L., Tang C., Wang R. (2019). Obesity Aggravates Acute Pancreatitis via Damaging Intestinal Mucosal Barrier and Changing Microbiota Composition in Rats. Sci. Rep..

[87] Krishna S.G., Hinton A., Oza V., Hart P.A., Swei E., El-Dika S., Stanich P.P., Hussan H., Zhang C., Conwell D.L. (2015). Morbid Obesity Is Associated With Adverse Clinical Outcomes in Acute Pancreatitis: A Propensity-Matched Study. Am. J. Gastroenterol..

[88] Dobszai D., Matrai P., Gyongyi Z., Csupor D., Bajor J., Eross B., Miko A., Szako L., Meczker A., Hagendorn R. (2019). Body-mass index correlates with severity and mortality in acute pancreatitis: A meta-analysis. World J. Gastroenterol..

[89] Cruz-Monserrate Z., Conwell D.L., Krishna S.G. (2016). The Impact of Obesity on Gallstone Disease, Acute Pancreatitis, and Pancreatic Cancer. Gastroenterol. Clin. N. Am..

[90] Martinez J., Johnson C.D., Sanchez-Paya J., de Madaria E., Robles-Diaz G., Perez-Mateo M. (2006). Obesity is a definitive risk factor of severity and mortality in acute pancreatitis: An updated meta-analysis. Pancreatology.

[91] Hong S., Qiwen B., Ying J., Wei A., Chaoyang T. (2011). Body mass index and the risk and prognosis of acute pancreatitis: A meta-analysis. Eur. J. Gastroenterol. Hepatol..

[92] Valdivielso P., Ramirez-Bueno A., Ewald N. (2014). Current knowledge of hypertriglyceridemic pancreatitis. Eur. J. Intern. Med..

[93] Zeng Y., Zhang W., Lu Y., Huang C., Wang X. (2014). Impact of hypertriglyceridemia on the outcome of acute biliary pancreatitis. Am. J. Med. Sci..

[94] Reddy S.K., Zhan M., Alexander H.R., El-Kamary S.S. (2013). Nonalcoholic fatty liver disease is associated with benign gastrointestinal disorders. World J. Gastroenterol..

[95] Yoon S.B., Lee I.S., Choi M.H., Lee K., Ham H., Oh H.J., Park S.H., Lim C.H., Choi M.G. (2017). Impact of Fatty Liver on Acute Pancreatitis Severity. Gastroenterol. Res. Pract..

[96] Nojgaard C. (2010). Prognosis of acute and chronic pancreatitis—A 30-year follow-up of a Danish cohort. Dan. Med. Bull..

[97] Miko A., Farkas N., Garami A., Szabo I., Vincze A., Veres G., Bajor J., Alizadeh H., Rakonczay Z., Vigh E. (2018). Preexisting Diabetes Elevates Risk of Local and Systemic Complications in Acute Pancreatitis: Systematic Review and Meta-analysis. Pancreas.

[98] Shen H.N., Lu C.L., Li C.Y. (2012). Effect of diabetes on severity and hospital mortality in patients with acute pancreatitis: A national population-based study. Diabetes Care.

[99] Nawaz H., O’Connell M., Papachristou G.I., Yadav D. (2015). Severity and natural history of acute pancreatitis in diabetic patients. Pancreatology.

[100] Kylanpaa M.L., Repo H., Puolakkainen P.A. (2010). Inflammation and immunosuppression in severe acute pancreatitis. World J. Gastroenterol..

[101] Martins F.D.O., Gomes B.C., Rodrigues A.S., Rueff J. (2017). Genetic Susceptibility in Acute Pancreatitis: Genotyping of GSTM1, GSTT1, GSTP1, CASP7, CASP8, CASP9, CASP10, LTA, TNFRSF1B, and TP53 Gene Variants. Pancreas.

[102] Ozhan G., Yanar H.T., Ertekin C., Alpertunga B. (2010). Polymorphisms in tumour necrosis factor alpha (TNFalpha) gene in patients with acute pancreatitis. Mediat. Inflamm..

[103] El-Ashmawy N.E., Khedr N.F., El-Bahrawy H.A., Hamada O.B. (2018). Suppression of inducible nitric oxide synthase and tumor necrosis factor-alpha level by lycopene is comparable to methylprednisolone in acute pancreatitis. Dig. Liver Dis..

[104] Exley A.R., Leese T., Holliday M.P., Swann R.A., Cohen J. (1992). Endotoxaemia and serum tumour necrosis factor as prognostic markers in severe acute pancreatitis. Gut.

[105] Paajanen H., Laato M., Jaakkola M., Pulkki K., Niinikoski J., Nordback I. (1995). Serum tumour necrosis factor compared with C-reactive protein in the early assessment of severity of acute pancreatitis. Br. J. Surg..

[106] Norman J., Franz M., Messina J., Riker A., Fabri P.J., Rosemurgy A.S., Gower W.R. (1995). Interleukin-1 receptor antagonist decreases severity of experimental acute pancreatitis. Surgery.

[107] Heresbach D., Letourneur J.P., Bahon I., Pagenault M., Guillou Y.M., Dyard F., Fauchet R., Malledant Y., Bretagne J.F., Gosselin M. (1998). Value of early blood Th-1 cytokine determination in predicting severity of acute pancreatitis. Scand. J. Gastroenterol..

[108] Chen C.C., Wang S.S., Lee F.Y., Chang F.Y., Lee S.D. (1999). Proinflammatory cytokines in early assessment of the prognosis of acute pancreatitis. Am. J. Gastroenterol..

[109] Matull W.R., Pereira S.P., O’Donohue J.W. (2006). Biochemical markers of acute pancreatitis. J. Clin. Pathol..

[110] Pastor C.M., Morel D.R., Vonlaufen A., Schiffer E., Lescuyer P., Frossard J.L. (2010). Delayed production of IL-18 in lungs and pancreas of rats with acute pancreatitis. Pancreatology.

[111] Soyalp M., Yalcin M., Oter V., Ozgonul A. (2017). Investigation of procalcitonin, IL-6, oxidative stress index (OSI) plasma and tissue levels in experimental mild and severe pancreatitis in rats. Bratisl. Lek. Listy.

[112] Jiang C.F., Shiau Y.C., Ng K.W., Tan S.W. (2004). Serum interleukin-6, tumor necrosis factor alpha and C-reactive protein in early prediction of severity of acute pancreatitis. J. Chin. Med Assoc..

[113] Li Y., Bai J., He B., Wang N., Wang H., Liu D. (2019). Weak association between the interleukin-8 rs4073 polymorphism and acute pancreatitis: A cumulative meta-analysis. BMC Med. Genet..

[114] Gross V., Andreesen R., Leser H.G., Ceska M., Liehl E., Lausen M., Farthmann E.H., Scholmerich J. (1992). Interleukin-8 and neutrophil activation in acute pancreatitis. Eur. J. Clin. Investig..

[115] Rau B., Steinbach G., Gansauge F., Mayer J.M., Grunert A., Beger H.G. (1997). The potential role of procalcitonin and interleukin 8 in the prediction of infected necrosis in acute pancreatitis. Gut.

[116] Dugernier T.L., Laterre P.F., Wittebole X., Roeseler J., Latinne D., Reynaert M.S., Pugin J. (2003). Compartmentalization of the inflammatory response during acute pancreatitis: Correlation with local and systemic complications. Am. J. Respir. Crit. Care Med..

[117] Wilson C., Heads A., Shenkin A., Imrie C. (1989). C-reactive protein, antiproteases and complement factors as objective markers of severity in acute pancreatitis. Br. J. Surg..

[118] Lelubre C., Anselin S., Zouaoui Boudjeltia K., Biston P., Piagnerelli M. (2013). Interpretation of C-reactive protein concentrations in critically ill patients. BioMed Res. Int..

[119] Cardoso F.S., Ricardo L.B., Oliveira A.M., Canena J.M., Horta D.V., Papoila A.L., Deus J.R. (2013). C-reactive protein prognostic accuracy in acute pancreatitis: Timing of measurement and cutoff points. Eur. J. Gastroenterol. Hepatol..

[120] Mayer A., McMahon M., Bowen M., Cooper E. (1984). C reactive protein: An aid to assessment and monitoring of acute pancreatitis. J. Clin. Pathol..

[121] Lavin M., Berger H.G., Warshan A.L., Buchler M.W., Carr-Locke D., Neoptolemos J.P., Russell C., Sarr M.G. (1998). Assessment of clinical severity and prognosis. The Pancreas.

[122] Büchler M., Malfertheiner P., Schoetensack C., Uhl W., Beger H.G. (1986). Sensitivity of antiproteases, complement factors and C-reactive protein in detecting pancreatic necrosis. Results of a prospective clinical study. Int. J. Pancreatol..

[123] Leese T., Shaw D., Holliday M. (1988). Prognostic markers in acute pancreatitis: Can pancreatic necrosis be predicted?. Ann. R. Coll. Surg. Engl..

[124] Miko A., Vigh E., Matrai P., Soos A., Garami A., Balasko M., Czako L., Mosdosi B., Sarlos P., Eross B. (2019). Computed Tomography Severity Index vs. Other Indices in the Prediction of Severity and Mortality in Acute Pancreatitis: A Predictive Accuracy Meta-analysis. Front. Physiol..

[125] Farkas N., Hanak L., Miko A., Bajor J., Sarlos P., Czimmer J., Vincze A., Godi S., Pecsi D., Varju P. (2019). A Multicenter, International Cohort Analysis of 1435 Cases to Support Clinical Trial Design in Acute Pancreatitis. Front. Physiol..

[126] Uhl W., Büchler M., Malfertheiner P., Martini M., Beger H.G. (1991). PMN-elastase in comparison with CRP, antiproteases, and LDH as indicators of necrosis in human acute pancreatitis. Pancreas.

[127] Pieri G., Agarwal B., Burroughs A.K. (2014). C-reactive protein and bacterial infection in cirrhosis. Ann. Gastroenterol. Q. Publ. Hell. Soc. Gastroenterol..

[128] Parniczky A., Lantos T., Toth E.M., Szakacs Z., Godi S., Hagendorn R., Illes D., Koncz B., Marta K., Miko A. (2019). Antibiotic therapy in acute pancreatitis: From global overuse to evidence based recommendations. Pancreatology.

[129] Al-Nawas B., Krammer I., Shah P.M. (1996). Procalcitonin in diagnosis of severe infections. Eur. J. Med. Res..

[130] Woo S.M., Noh M.H., Kim B.G., Hsing C.T., Han J.S., Ryu S.H., Seo J.M., Yoon H.A., Jang J.S., Choi S.R. (2011). Comparison of serum procalcitonin with Ranson, APACHE-II, Glasgow and Balthazar CT severity index scores in predicting severity of acute pancreatitis. Korean J. Gastroenterol..

[131] Purkayastha S., Chow A., Athanasiou T., Cambaroudis A., Panesar S., Kinross J., Tekkis P., Darzi A. (2006). Does serum procalcitonin have a role in evaluating the severity of acute pancreatitis? A question revisited. World J. Surg..

[132] Neoptolemos J.P. (2001). Procalcitonin strip test in the early detection of severe acute pancreatitis. Br. J. Surg..

[133] Dias B.H., Rozario A.P., Olakkengil S.A., Anirudh V. (2015). Procalcitonin Strip Test as an Independent Predictor in Acute Pancreatitis. Indian J. Surg..

[134] Mofidi R., Suttie S.A., Patil P.V., Ogston S., Parks R.W. (2009). The value of procalcitonin at predicting the severity of acute pancreatitis and development of infected pancreatic necrosis: Systematic review. Surgery.

[135] Siriwardena A.K., Jegatheeswaran S., Mason J.M., Baltatzis M., Chan A., Sheen A.J., O’Reilly D., Jamdar S., Deshpande R., de Liguori Carino N. (2019). PROCalcitonin-based algorithm for antibiotic use in Acute Pancreatitis (PROCAP): Study protocol for a randomised controlled trial. Trials.

[136] Lee W.L., Downey G.P. (2001). Leukocyte elastase: Physiological functions and role in acute lung injury. Am. J. Respir. Crit. Care Med..

[137] Dominguez-Munoz J.E., Villanueva A., Larino J., Mora T., Barreiro M., Iglesias-Canle J., Iglesias-Garcia J. (2006). Accuracy of plasma levels of polymorphonuclear elastase as early prognostic marker of acute pancreatitis in routine clinical conditions. Eur. J. Gastroenterol. Hepatol..

[138] Ikei S., Ogawa M., Yamaguchi Y. (1998). Blood concentrations of polymorphonuclear leucocyte elastase and interleukin-6 are indicators for the occurrence of multiple organ failures at the early stage of acute pancreatitis. J. Gastroenterol. Hepatol..

[139] Mackman N. (2009). The many faces of tissue factor. J. Thromb. Haemost..

[140] Andersson E., Axelsson J., Eckerwall G., Ansari D., Andersson R. (2010). Tissue factor in predicted severe acute pancreatitis. World J. Gastroenterol..

[141] Ou Z.B., Miao C.M., Ye M.X., Xing D.P., He K., Li P.Z., Zhu R.T., Gong J.P. (2017). Investigation for role of tissue factor and blood coagulation system in severe acute pancreatitis and associated liver injury. Biomed. Pharmacother..

[142] Babitt J.L., Huang F.W., Wrighting D.M., Xia Y., Sidis Y., Samad T.A., Campagna J.A., Chung R.T., Schneyer A.L., Woolf C.J. (2006). Bone morphogenetic protein signaling by hemojuvelin regulates hepcidin expression. Nat. Genet..

[143] Kulaksiz H., Fein E., Redecker P., Stremmel W., Adler G., Cetin Y. (2008). Pancreatic b-cells express hepcidin, an iron-uptake regulatory peptide. J. Endocrinol..

[144] Arabul M., Celik M., Aslan O., Torun S., Beyazit Y., Alper E., Kandemir A., Unsal B. (2013). Hepcidin as a predictor of disease severity in acute pancreatitis: A single center prospective study. Hepatogastroenterology.

[145] Katan M., Muller B., Christ-Crain M. (2008). Copeptin: A new and promising diagnostic and prognostic marker. Crit. Care.

[146] Isman F.K., Zulfikaroglu B., Isbilen B., Ozalp N., Ozmen M.M., Bilgic I., Koc M. (2013). Copeptin is a predictive biomarker of severity in acute pancreatitis. Am. J. Emerg. Med..

[147] Nebiker C.A., Staubli S., Schafer J., Bingisser R., Christ-Crain M., Dell-Kuster S., Mueller C., Scamardi K., Viehl C.T., Kolleth D. (2018). Cortisol Outperforms Novel Cardiovascular, Inflammatory, and Neurohumoral Biomarkers in the Prediction of Outcome in Acute Pancreatitis. Pancreas.

[148] Ida S., Fujimura Y., Hirota M., Imamura Y., Ozaki N., Suyama K., Hashimoto D., Ohmuraya M., Tanaka H., Takamori H. (2009). Significance of endothelial molecular markers in the evaluation of the severity of acute pancreatitis. Surg. Today.

[149] Milnerowicz S., Milnerowicz H., Nabzdyk S., Jablonowska M., Grabowski K., Tabola R. (2013). Plasma endothelin-1 levels in pancreatic inflammations. Adv. Clin. Exp. Med..

[150] Bennett J., Cooper D., Balakrishnan A., Rhodes M., Lewis M. (2006). Is there a role for serum endothelin in predicting the severity of acute pancreatitis?. Hepatobiliary Pancreat. Dis. Int..

[151] Nukarinen E., Lindstrom O., Kuuliala K., Kylanpaa L., Pettila V., Puolakkainen P., Kuuliala A., Hamalainen M., Moilanen E., Repo H. (2016). Association of Matrix Metalloproteinases -7, -8 and -9 and TIMP -1 with Disease Severity in Acute Pancreatitis. A Cohort Study. PLoS ONE.

[152] Wereszczynska-Siemiatkowska U., Siemiatkowski A., Swidnicka-Siergiejko A., Mroczko B., Dabrowski A. (2015). The imbalance between matrix metalloproteinase 9 and tissue inhibitor of metalloproteinase 1 in acute pancreatitis. Z. Gastroenterol..

[153] Guo J., Xue P., Yang X.N., Liu X.B., Huang W., Xia Q. (2012). Serum matrix metalloproteinase-9 is an early marker of pancreatic necrosis in patients with severe acute pancreatitis. Hepatogastroenterology.

[154] Chen P., Yuan Y., Wang S., Zhan L., Xu J. (2006). Serum matrix metalloproteinase 9 as a marker for the assessment of severe acute pancreatitis. Tohoku J. Exp. Med..

[155] Kim H.J., Lee H.W. (2013). Important predictor of mortality in patients with end-stage liver disease. Clin. Mol. Hepatol..

[156] Holliday M.P., Shaw D., Thomas W.M., Leese T. (1989). Threshold for albumin as a prognostic marker in acute pancreatitis. Br. J. Surg..

[157] Gonzalvez-Gasch A., de Casasola G.G., Martin R.B., Herreros B., Guijarro C. (2009). A simple prognostic score for risk assessment in patients with acute pancreatitis. Eur. J. Intern. Med..

[158] Hong W., Lin S., Zippi M., Geng W., Stock S., Basharat Z., Cheng B., Pan J., Zhou M. (2017). Serum Albumin Is Independently Associated with Persistent Organ Failure in Acute Pancreatitis. Can. J. Gastroenterol. Hepatol..

[159] Huai J., Shao Y., Sun X., Jin Y., Wu J., Huang Z. (2012). Melatonin ameliorates acute necrotizing pancreatitis by the regulation of cytosolic Ca2+ homeostasis. Pancreatology.

[160] Gutierrez-Jimenez A.A., Castro-Jimenez E., Lagunes-Cordoba R. (2014). Total serum calcium and corrected calcium as severity predictors in acute pancreatitis. Rev. Gastroenterol. Mex..

[161] Yu S., Wu D., Jin K., Yin L., Fu Y., Liu D., Zhang L., Yu X., Xu J. (2019). Low Serum Ionized Calcium, Elevated High-Sensitivity C-Reactive Protein, Neutrophil-Lymphocyte Ratio, and Body Mass Index (BMI) Are Risk Factors for Severe Acute Pancreatitis in Patients with Hypertriglyceridemia Pancreatitis. Med Sci. Monit. Int. Med J. Exp. Clin. Res..

[162] Whitcomb D.C., Lowe M.E. (2007). Human pancreatic digestive enzymes. Dig. Dis. Sci..

[163] Wu H.C., Wang H.P., Chen C.C., Wu C.H., Liu T.H., Wang C.H., Shih L.N., Liao W.C. (2019). Urinary trypsinogen-2 level and local complications of acute pancreatitis. J. Gastroenterol. Hepatol..

[164] Yasuda H., Kataoka K., Takeyama Y., Takeda K., Ito T., Mayumi T., Isaji S., Mine T., Kitagawa M., Kiriyama S. (2019). Usefulness of urinary trypsinogen-2 and trypsinogen activation peptide in acute pancreatitis: A multicenter study in Japan. World J. Gastroenterol..

[165] Deng L., Wang L., Yong F., Xiong J., Jin T., De La Iglesia-Garcia D., Bharucha S., Altaf K., Huang W., Xia Q. (2015). Prediction of the severity of acute pancreatitis on admission by carboxypeptidase-B activation peptide: A systematic review and meta-analysis. Clin. Biochem..

[166] Goyal H., Awad H., Hu Z.-D. (2017). Prognostic value of admission red blood cell distribution width in acute pancreatitis: A systematic review. Ann. Transl. Med..

[167] Gravito-Soares M., Gravito-Soares E., Gomes D., Almeida N., Tome L. (2018). Red cell distribution width and red cell distribution width to total serum calcium ratio as major predictors of severity and mortality in acute pancreatitis. BMC Gastroenterol..

[168] Zhou H., Mei X., He X., Lan T., Guo S. (2019). Severity stratification and prognostic prediction of patients with acute pancreatitis at early phase: A retrospective study. Medicine.

[169] Harrison D.A., D’Amico G., Singer M. (2007). The Pancreatitis Outcome Prediction (POP) Score: A new prognostic index for patients with severe acute pancreatitis. Crit. Care Med..

[170] Wu B.U., Johannes R.S., Sun X., Conwell D.L., Banks P.A. (2009). Early changes in blood urea nitrogen predict mortality in acute pancreatitis. Gastroenterology.

[171] Gan S.I., Romagnuolo J. (2004). Admission hematocrit: A simple, useful and early predictor of severe pancreatitis. Dig. Dis. Sci..

[172] Parsa N., Faghih M., Garcia Gonzalez F., Moran R.A., Kamal A., Jalaly N.Y., Al-Grain H., Akshintala V.S., Makary M.A., Khashab M.A. (2019). Early Hemoconcentration Is Associated With Increased Opioid Use in Hospitalized Patients with Acute Pancreatitis. Pancreas.

[173] Baillargeon J.-D., Orav J., Ramagopal V., Tenner S., Banks P.A. (1998). Hemoconcentration as an early risk factor for necrotizing pancreatitis. Am. J. Gastroenterol..

[174] Brown A., Orav J., Banks P.A. (2000). Hemoconcentration is an early marker for organ failure and necrotizing pancreatitis. Pancreas.

[175] Lankisch P.G., Mahlke R., Blum T., Bruns A., Bruns D., Maisonneuve P., Lowenfels A.B. (2001). Hemoconcentration: An early marker of severe and/or necrotizing pancreatitis? A critical appraisal. Am. J. Gastroenterol..

[176] Talamini G., Uomo G., Pezzilli R., Rabitti P.G., Billi P., Bassi C., Cavallini G., Pederzoli P. (1999). Serum creatinine and chest radiographs in the early assessment of acute pancreatitis. Am. J. Surg..

[177] Muddana V., Whitcomb D.C., Khalid A., Slivka A., Papachristou G.I. (2009). Elevated serum creatinine as a marker of pancreatic necrosis in acute pancreatitis. Am. J. Gastroenterol..

[178] Shearman C.P., Gosling P., Walker K.J. (1989). Is low proteinuria an early predictor of severity of acute pancreatitis?. J. Clin. Pathol..

[179] Zuidema M.J., van Santvoort H.C., Besselink M.G., van Ramshorst B., Boerma D., Timmer R., Bollen T.L., Weusten B.L. (2014). The predictive value of proteinuria in acute pancreatitis. Pancreatology.

[180] Orfanos S.E., Kotanidou A., Glynos C., Athanasiou C., Tsigkos S., Dimopoulou I., Sotiropoulou C., Zakynthinos S., Armaganidis A., Papapetropoulos A. (2007). Angiopoietin-2 is increased in severe sepsis: Correlation with inflammatory mediators. Crit. Care Med..

[181] Whitcomb D.C., Muddana V., Langmead C.J., Houghton F.D., Guenther A., Eagon P.K., Mayerle J., Aghdassi A.A., Weiss F.U., Evans A. (2010). Angiopoietin-2, a regulator of vascular permeability in inflammation, is associated with persistent organ failure in patients with acute pancreatitis from the United States and Germany. Am. J. Gastroenterol..

[182] Franco-Pons N., Casas J., Fabrias G., Gea-Sorli S., de-Madaria E., Gelpi E., Closa D. (2013). Fat necrosis generates proinflammatory halogenated lipids during acute pancreatitis. Ann. Surg..

[183] de-Madaria E., Molero X., Bonjoch L., Casas J., Cardenas-Jaen K., Montenegro A., Closa D. (2018). Oleic acid chlorohydrin, a new early biomarker for the prediction of acute pancreatitis severity in humans. Ann. Intensive Care.

[184] Harris S., Nadkarni N.A., Naina H.V., Vege S.S. (2013). Splanchnic vein thrombosis in acute pancreatitis: A single-center experience. Pancreas.

[185] Lisman T., Porte R.J. (2010). Activation and regulation of hemostasis in acute liver failure and acute pancreatitis. Semin. Thromb. Hemost..

[186] Radenkovic D., Bajec D., Ivancevic N., Milic N., Bumbasirevic V., Jeremic V., Djukic V., Stefanovic B., Stefanovic B., Milosevic-Zbutega G. (2009). D-dimer in acute pancreatitis: A new approach for an early assessment of organ failure. Pancreas.

[187] Wan J., Yang X., He W., Zhu Y., Zhu Y., Zeng H., Liu P., Xia L., Lu N. (2019). Serum D-dimer levels at admission for prediction of outcomes in acute pancreatitis. BMC Gastroenterol..

[188] Ou X., Cheng Z., Liu T., Tang Z., Huang W., Szatmary P., Zheng S., Sutton R., Toh C.H., Zhang N. (2015). Circulating Histone Levels Reflect Disease Severity in Animal Models of Acute Pancreatitis. Pancreas.

[189] Liu T., Huang W., Szatmary P., Abrams S.T., Alhamdi Y., Lin Z., Greenhalf W., Wang G., Sutton R., Toh C.H. (2017). Accuracy of circulating histones in predicting persistent organ failure and mortality in patients with acute pancreatitis. Br. J. Surg..

[190] Xu J., Zhang X., Pelayo R., Monestier M., Ammollo C.T., Semeraro F., Taylor F.B., Esmon N.L., Lupu F., Esmon C.T. (2009). Extracellular histones are major mediators of death in sepsis. Nat. Med..

[191] Kang R., Lotze M.T., Zeh H.J., Billiar T.R., Tang D. (2014). Cell death and DAMPs in acute pancreatitis. Mol. Med..

[192] Biberci Keskin E., Ince A.T., Sumbul Gultepe B., Koker I.H., Senturk H. (2019). The relationship between serum histon levels and the severity of acute pancreatitis. Turk. J. Gastroenterol..

[193] Liu J., Wang G., Liu Y., Huang L., Xu X., Wang J. (2019). Effects of Somatostatin Combined with Pantoprazole on Serum C-Reactive Protein and Intercellular Adhesion Molecule-1 in Severe Acute Pancreatitis. J. Coll. Physicians Surg. Pak..

[194] Zhu H.H., Jiang L.L. (2012). Serum inter-cellular adhesion molecule 1 is an early marker of diagnosis and prediction of severe acute pancreatitis. World J. Gastroenterol..

[195] Petricoin E.F., Zoon K.C., Kohn E.C., Barrett J.C., Liotta L.A. (2002). Clinical proteomics: Translating benchside promise into bedside reality. Nat. Rev. Drug Discov..

[196] Papachristou G.I., Malehorn D.E., Lamb J., Slivka A., Bigbee W.L., Whitcomb D.C. (2007). Serum proteomic patterns as a predictor of severity in acute pancreatitis. Pancreatology.

[197] Fetaud V., Frossard J.L., Farina A., Pastor C.M., Buhler L., Dumonceau J.M., Hadengue A., Hochstrasser D.F., Lescuyer P. (2008). Proteomic profiling in an animal model of acute pancreatitis. Proteomics.

[198] Sandstrom A., Andersson R., Segersvard R., Lohr M., Borrebaeck C.A., Wingren C. (2012). Serum proteome profiling of pancreatitis using recombinant antibody microarrays reveals disease-associated biomarker signatures. Proteom. Clin. Appl..

[199] Garcia-Hernandez V., Sanchez-Bernal C., Sarmiento N., Viana R.A., Ferreira L., Perez N., Calvo J.J., Sanchez-Yague J. (2012). Proteomic analysis of the soluble and the lysosomal+mitochondrial fractions from rat pancreas: Implications for cerulein-induced acute pancreatitis. Biochim. Biophys. Acta.

[200] Ma C., Tian B., Wang J., Yang G.-J., Pan C.-S., Lu J.-P. (2012). Metabolic characteristics of acute necrotizing pancreatitis and chronic pancreatitis. Mol. Med. Rep..

[201] Xiao H., Huang J.-h., Zhang X.-w., Ahmed R., Xie Q.-l., Li B., Zhu Y.-m., Cai X., Peng Q.-h., Qin Y.-h. (2017). Identification of potential diagnostic biomarkers of acute pancreatitis by serum metabolomic profiles. Pancreatology.

[202] van Brunschot S., van Grinsven J., van Santvoort H.C., Bakker O.J., Besselink M.G., Boermeester M.A., Bollen T.L., Bosscha K., Bouwense S.A., Bruno M.J. (2018). Endoscopic or surgical step-up approach for infected necrotising pancreatitis: A multicentre randomised trial. Lancet.

